# Fractionated Total Body Irradiation on an Infant Using Tomotherapy

**DOI:** 10.7759/cureus.28143

**Published:** 2022-08-18

**Authors:** Usha Abraham, Tino Romaguera, Ranjini Tolakanahalli, Alonso N Gutierrez, Matthew Hall

**Affiliations:** 1 Radiation Oncology, Miami Cancer Institute, Miami, USA; 2 Radiation Oncology, Herbert Wertheim College of Medicine, Miami, USA; 3 Department of Radiation Oncology, Miami Cancer Institute, Miami, USA

**Keywords:** mvct, mean lung dose, tomotherapy, acute lymphoblastic leukemia, total body irradiation

## Abstract

Total body irradiation (TBI) is used with chemotherapy to induce immunosuppression for hematopoietic cell transplantation and is often administered using lead blocks to minimize lung dose in adults and children. This technique is challenging in infants and young children. A 13-month-old female with acute lymphoblastic leukemia (ALL) was treated with fractionated TBI to a dose of 12 Gy in eight fractions delivered twice daily. Multiple TBI techniques for delivering treatment were considered. Ultimately, treatment using helical tomotherapy was selected in order to spare and accurately quantify the dose to the lung, meet lung dose constraints, and ensure adequate TBI dose coverage. With anesthesia, this technique provided a comfortable and reproducible set-up for the young child. The treatment plan was delivered with intensity-modulated radiotherapy, where 96.4% of the target volume received a prescription dose with a total beam-on time of 16.8 minutes. The mean lung dose was 7.7 Gy for a total lung volume of 245cc. This report describes the challenges faced during the treatment planning and delivery, and how they were resolved.

## Introduction

At our institution, two total body irradiation (TBI) techniques using a conventional linear accelerator have been commissioned. The primary technique uses two parallel-opposed beams in the anterior-posterior orientation (AP/PA) with the coronal plane of the patient [1]. For this treatment, the patient stands upright within a TBI positioning stand at an extended distance of 422 cm source to patient midline; the lung blocks are positioned 30 cm from the patient’s midline. The second technique requires the patient in the lateral decubitus or supine position on a gurney at 422 cm to the patient’s midline [1]. With this position, the lung blocks are placed at 70 cm from patient’s midline due to the positioning location of the block holder. Both techniques require the patient to maintain their position for accurate treatment and lung blocking. In young patients, this presents a challenge due to treatment compliance and the smaller size of the lungs. A third method for total body irradiation treatment was commissioned on the Radixact® (Accuray Incorporated, Sunnyvale, California, United States) unit using a helical tomotherapy approach. This report describes the treatment delivery of fractionated TBI using helical tomotherapy with pediatric anesthesia, which enabled delivery of the prescribed dose with appropriate sparing of the lungs [2]. This technique may serve as an equivalent technological alternative to consider when patients are young and lung sizes are small for TBI using two-dimensional treatment planning techniques.

## Case presentation

The patient is a 13-month-old female who presented to the emergency department at an age of eight months with bruising and swelling of the eyelids and general malaise. Laboratory studies demonstrated hemoglobin of 4.3, thrombocytopenia with platelets of 9,000, white blood cell (WBC) count of 236,000, and lactate dehydrogenase (LDH) of 1643. On physical exam, hepatosplenomegaly was noted. Flow cytometry was consistent with pre-B acute lymphoblastic leukemia (ALL), with approximately 85% of cells consistent with leukemia. On molecular testing, no evidence of the Philadelphia chromosome or KMT2A gene rearrangement was identified. The initial cerebrospinal fluid analysis demonstrated 28 WBCs, consistent with CNS3 disease. The patient initiated induction chemotherapy as per Children’s Oncology Group study AALL15P1. At the end of induction, the patient was not in remission with 13% residual bone marrow involvement. The patient initiated induction intensification; after one cycle, the bone marrow remained positive at 10.5%. The patient then received re-induction therapy with blinatumomab. After one cycle, the patient achieved remission and was minimal residual disease (MRD) negative.

The patient was evaluated by the bone marrow transplant team and hematopoietic cell transplantation (HSCT) was recommended. A donor match failed to identify matched sibling and unrelated donors; the best match was an unrelated cord blood donor with a 6/8 human leukocyte antigens (HLA) match. For the unrelated cord blood transplant (UCBT), the recommended conditioning regimen was fludarabine, fractionated TBI to 12 Gy in eight fractions delivered twice daily, and cyclophosphamide. The total dose for TBI was selected with consultation from specialists at other bone marrow transplant centers. Because rapid clearance of the CNS3 disease had been observed, no cranial boost was planned. The patient continued blinatumomab leading into HSCT.

**Figure 1:**
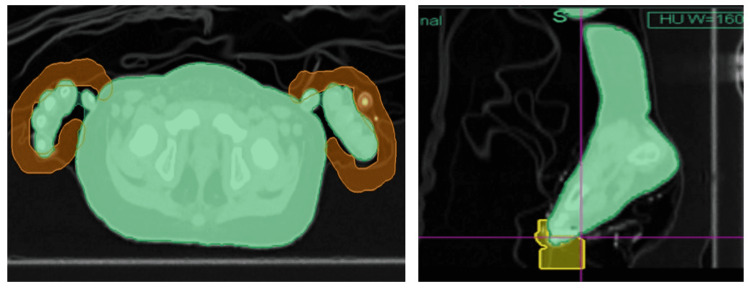
Axial pelvic slice (left) and sagittal foot slice (right) are shown of the pediatric patient. Region of Interest (ROI) with density override created around the fingers and toes to prevent under-dosing of these regions due to uncertainty in the reproducibility.

Due to the patient’s young age and small lung sizes, helical tomotherapy on Radixact® was selected for treatment. This was decided by the clinical team in order to maintain adequate coverage of the prescription dose to the target volume, meet lung dose constraints, and ensure treatment delivery accuracy [2,3]. CT simulation was performed to deliver treatment in a supine position with VacQfix™ Vacuum Cushions (QFix, Avondale, Pennsylvania, United States) and mask for immobilization. For treatment compliance, TBI delivery was planned with total intravenous anesthesia using propofol. The patient was undressed before scanning and placed on chuck instead of sheets for reproducibility during treatment. The arms were placed very close to the side of the body and fingers were stretched out (Figure 1). 

The patient was marked with Tegaderm™ (3M Company, Saint Paul, Minnesota, United States). No tattoos were used due to the age of the patient. A CT scan of the entire body was acquired with a 3 mm slice thickness [4]. For planning, the right and left lungs, total lungs, kidneys, spinal cord, and eyes were contoured and designated as organs at risk (OARs). The planning target volume (PTV) consisted of the entire body with 3 mm cropped off of the skin and the lungs. The patient, while under anesthesia, was a shallow breather and thereby had minimal variation in the chest region. Since the tips of the fingers and toes may not be reproducible and likely to be out of the radiation field, a 5-10 mm virtual bolus was created around them and was given a density override of 1 g/cc (Figure 1). The treatment prescription was at least 95% of the PTV receiving 12 Gy in eight fractions, two fractions per day (BID). The main OAR constraint was the mean dose to the lungs to be less than 8 Gy to prevent pneumonitis. Constraints were also placed on the kidneys and spinal cord to reduce hot spots in these structures.

**Figure 2:**
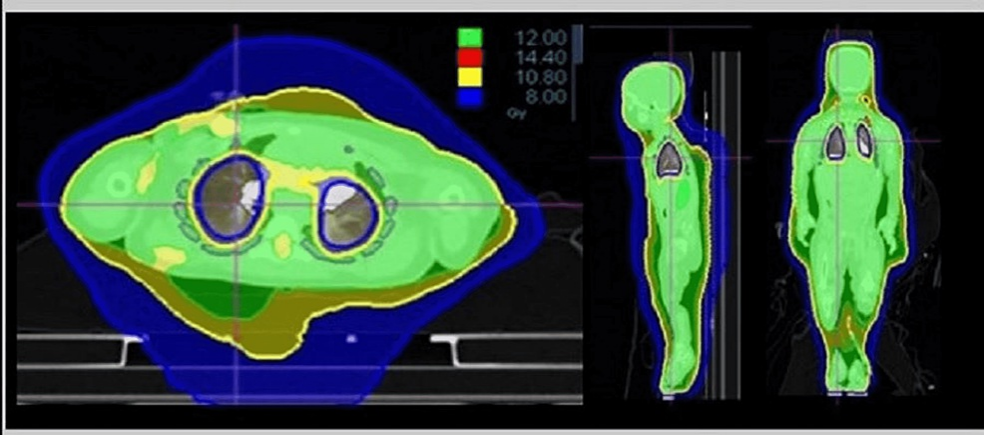
The 3D dose distribution of the helical tomotherapy TBI plan is shown in axial (left), sagittal (middle), and coronal (right) planes. The prescription dose of 12Gy is represented by the green area. Isodose level is displayed outside the body. TBI: total body irradiation

**Figure 3:**
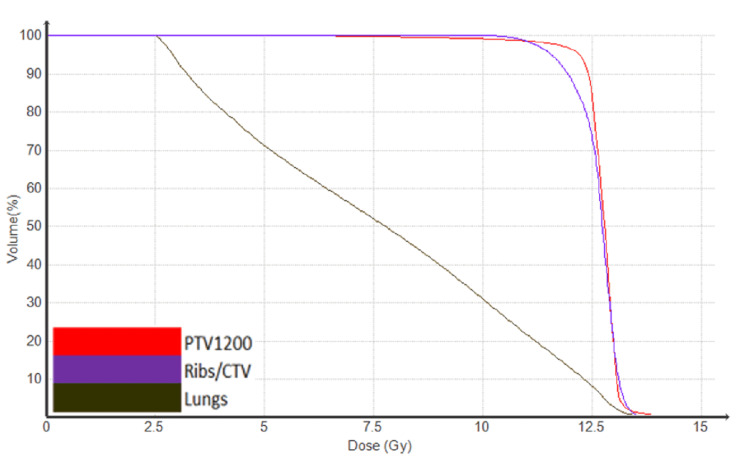
Dose volume histogram (DVH) to targets and lungs CTV: clinical target volume; PTV: planning target volume

In this plan, 96.4% of the target (PTV) received at least the prescription dose of 12 Gy while 93% of the chest wall, which is included in the PTV, also received the prescription dose. The mean total lung dose obtained was 7.7 Gy. The dose to the region of interest (ROI) is presented in Table 1 (Figure 2, 3). Regarding the treatment plan parameters, a dynamic field width of 5 cm, pitch of 0.25, and a modulator factor (MF) of 2.0 were used [5]. A 3D conformal plan was also developed in order to reduce the treatment time, but dosimetric goals could not be achieved.

**Table 1:** Select dose volume histogram (DVH) parameters for the target volumes and organs at risk (OARs) PTV: planning target volume; L: left; R: right; CW: chest wall; CTV: clinical target volume

Organs	Dose(Gy)	Dose(%)		Volume (cc)		Volume (%)
PTV1200	12.00	100.00		8838.30		96.40
CTV_Ribs_1200	12.00	100.00		60.87		88.70
L_CW	12.00	100.00		161.93		93.30
R_CW	12.00	100.00		170.28		93.30
Lungs_L	7.88	65.60		46.75		50.00
Lung_R	7.64	63.70		74.25		50.00
Lungs	7.74	64.50		121.00		50.00
Kidney_L	13.38	111.50		1.00		1.90
Kidney_R	13.24	110.30		1.00		2.30
Spinal Cord	13.25	110.5		0.03		0.1
Organs	Constraints					
PTV1200	D98% = 11.4Gy		D2% = 13.3Gy		Mean Dose = 12.7Gy	
Ribs/CW	D98% = 11.5Gy		D2% = 13.3Gy		Mean Dose = 12.6Gy	
Lungs	D99% = 2.6Gy		V8Gy = 48%		Mean Dose = 7.7Gy	

Prior to treatment, three quality assurance (QA) plans were created within the delivery platform to verify the planned dose at the level of the head, chest, and umbilicus/pelvis regions and measured using an ion chamber and a diode array ArcCheck (SunNuclear, Mirion Technologies, Inc., Atlanta, Georgia, United States). Greater than 99% of the points in the QA plans were within tolerance for plans evaluated with local Gamma criteria of 3%/3 mm and a threshold of 10%. The absolute dose measured using an ion chamber was within 2% of the predicted dose for each of the QA plans.

**Figure 4:**
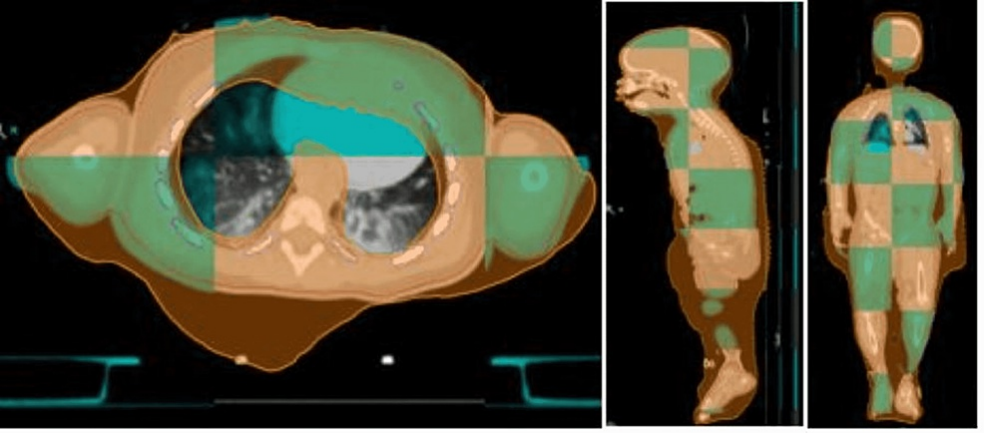
Megavoltage CT (MVCT) - planning CT image registration in axial (left), sagittal (middle), and coronal (right) view. The MVCT image shows the body is within the 95% isodose colorwash displayed in orange.

Image-guided radiation therapy (IGRT) [6] using Megavoltage CT (MVCT) scan of the entire body was performed to ensure accurate positioning of the patient (Figure 4). The treatment beam on time was 16.8 minutes. The time spent in the vault for an entire treatment procedure was between 1.5-2 hours.

In vivo dosimetry was performed using optically stimulated luminescence dosimeters (OSLDs) placed under 0.5 cm bolus to measure the dose to the skin at various regions of the body. The results are shown in Table 2. Bolus was used as buildup material to provide electronic equilibrium for the 6 MV beam. This confirmed the dose delivered to these sites was within tolerance. The OSLDs placed on the finger and toes were within 10% of prescription dose.

**Table 2:** In vivo dosimetry, using OSLD measurements at various regions of the body were made for a single treatment session of the TBI OSLD: optically stimulated luminescence dosimeter; TBI: total body irradiation

In-vivo dosimetry - OSLD results			
Site	Measured dose	Mean prescription dose	% difference
Chest	1.58	1.50	5.3
Umbilicus	1.54	1.50	2.7
Right Foot (toes)	1.45	1.50	-3.3
Left Knee	1.59	1.50	6.0
Left Hand (fingers)	1.62	1.50	8.0

After completion of TBI, the patient underwent stem cell infusion as planned. Engraftment was documented on Day +18, and she was discharged from the hospital on Day +49. At the time of the last follow-up, the patient was alive with no evidence of disease five months after HSCT. Bone marrow biopsy on Day +140 following HSCT demonstrated no evidence of residual leukemia (MRD negative) with 100% donor chimerism. At the last follow-up, there was no clear evidence of graft versus host disease, although the patient was receiving tacrolimus. 

## Discussion

TBI is generally recommended as part of the conditioning regimen for HSCT in patients with ALL. The primary indications for HSCT in ALL are first remission with poor risk features, including Philadelphia chromosome-positive or having disease that does not reach remission after initial induction chemotherapy, and for relapsed disease [7]. Typical dosing regimens range from 12-13.75 Gy delivered using two to three fractions per day. In addition to ensuring adequately homogeneous dosing of the entire body, treatment also entails a reduction in dose to the lungs, with an objective to reduce the mean lung dose to <8 Gy [8-10], although some groups have recommended <7 Gy for further risk reduction [11]. While the number of ALL patients that require HSCT has decreased over time with improvements in therapeutic options, selected patients still must receive HSCT for high-risk or relapsed disease, as in this case report. In children, TBI delivery is made significantly more difficult due to treatment compliance and with techniques that were generally designed for patients who were older, larger, and occasionally more compliant. 

Reducing the dose to critical structures to decrease the acute and long-term toxicities was essential due to the age of the patient. The volume of the lungs of the infant was 245 cc, while that of an adult is usually 2000-3000 cc. Applying necessary margins to the lungs, the dimension of Cerrobend lung block needed for an AP/PA open field with the conventional linac would be approximately 0.5 cm X 5.5 cm assuming the patient is in lateral decubitus position [1]. It would be very challenging to reduce the mean lung dose to less than 8 Gy while ensuring at least 90% of the chest wall/ribs receive the prescription dose using this setup for treatment. Helical tomotherapy was preferred over the conventional linear accelerator [2] due to the need for a patient-specific conformal plan to provide better dose sculpting to the critical structures and optimal dose coverage over an elongated target [12,13]. A sharp dose gradient is obtained by intensity modulated delivery of radiation by the helical delivery using 64 MLCs and 51 gantry projections per rotation.

For the tomotherapy plan, the chest wall/ribs were termed CTV and given an overlap priority of 1 while the PTV was given an overlap priority of 2. ROIs with inner margins of 3 mm and 8 mm into the lungs were used as optimization structures to minimize lung dose. The quality of the plan was improved by varying the parameters like pitch, modulation factor, and the importance of the specific optimization structures (ROI) like Lung-3 mm and Lungs-8 mm [4]. The highly conformal tomotherapy plan was able to carve out the dose to the lungs while delivering the required dose to the surrounding chest wall as represented in Table 1. The coverage was excellent considering the size of the patient and the volume of the lungs [1,2].

The virtual bolus of around 1 cm with density override of 1 g/cc was extended inferiorly around the toes and heel in case the leg which was curved during simulation were to be extended during treatment causing under-dosing of these regions. Similarly, a 5-10 mm thickness virtual bolus was added around the fingers to account for positioning variability (figure 1). Extending the CT scan a couple of slices inferiorly beyond the anatomy of the patient is recommended in case the virtual bolus has to be added during planning. The tips of the fingers and toes received an additional dose from the additional fluence planned due to the virtual bolus. This was accepted since there weren’t any critical structures in this region that would be affected by the excess dose. OSLDs were placed in these regions to verify the dose to the surface.

For IGRT, the capability of taking MVCT scans of the entire length of the patient and registering it to the planning CT is an exclusive feature of helical tomotherapy (Figure 4). Since the positioning marks on the patient were lost, the superior-inferior positioning was not accurate; this created a longitudinal shift of 4.7 cm for the first treatment. The patient was repositioned to correct for setup issues and a second MVCT scan was taken. New setup marks were drawn on the patient. A second MVCT had to be taken in two out of the eight treatments. The shifts after image registration for the rest of the treatments were less than 8 mm. The patient had gained 1 Kg weight between simulation and treatment, which provided a challenge during setup and image registration. Since babies have growth spurts it may be desirable to decrease the duration between simulation and treatment. Distension of the abdomen resulted in a discrepancy between MVCT and planning CT anteriorly, which was considered acceptable as there was no bone in the concerned region and was encompassed by the 95% isodose line (Figure 4).

Since the patient was only 82 cm in length, it was possible to treat the entire body using a single plan in head first supine (HFS) position in 16.8 minutes [2-4]. Another advantage of treatment on the Radixact was that the patient had a more comfortable setup as they lie supine with arms at the side in an immobilization device. Due to the complexity of the treatment, it required the presence of the anesthesia and nursing team. In addition, the pediatric radiation oncologist and physicist were present to confirm the setup and image registration before each treatment.

## Conclusions

Helical tomotherapy via the Radixact® platform is a useful treatment option for TBI in very young patients who cannot readily receive two-dimensional techniques. Supine position is more comfortable and highly reproducible, especially for cases treated under anesthesia. In this case, the patient was 13 months old and TBI using tomotherapy enabled a conformal and homogenous treatment plan, and reliable delivery. Target coverage exceeded expected dose volume coverage and mean lung dose below tolerance, resulting in an acceptable risk of pneumonitis. The total treatment time using this technique was significantly less than using conventional techniques when considering that electron treatments for the chest wall boost were not required. IGRT with MVCT helped to ensure accurate patient positioning and accurate delivery of the prescribed treatment plan. 

## References

[1] (1986). The Physical Aspects Of Total And Half Body Photon Irradiation.

[2] Konishi T, Ogawa H, Najima Y (2020). Safety of total body irradiation using intensity-modulated radiation therapy by helical tomotherapy in allogeneic hematopoietic stem cell transplantation: a prospective pilot study. J Radiat Res.

[3] Gruen A, Ebell W, Wlodarczyk W (2013). Total body irradiation (TBI) using helical tomotherapy in children and young adults undergoing stem cell transplantation. Radiat Oncol.

[4] Sresty NV, Gudipudi D, Krishnam Raju A, Anil Kumar T, Lakshmi VR, Srikanth G, Narasimha M (2021). Total body irradiation of bone marrow transplant using helical TomoTherapy with a focus on the quality of dose contribution at junction target volumes. Strahlenther Onkol.

[5] Shimizu H, Sasaki K, Kubota T (2018). Interfacility variation in treatment planning parameters in tomotherapy: field width, pitch, and modulation factor. J Radiat Res.

[6] Yartsev S, Kron T, Van Dyk J (2007). Tomotherapy as a tool in image-guided radiation therapy (IGRT): theoretical and technological aspects. Biomed Imaging Interv J.

[7] Wong JY, Filippi AR, Dabaja BS, Yahalom J, Specht L (2018). Total body irradiation: guidelines from the international lymphoma radiation oncology group (ILROG). Int J Radiat Oncol Biol Phys.

[8] Sampath S, Schultheiss TE, Wong J (2005). Dose response and factors related to interstitial pneumonitis after bone marrow transplant. Int J Radiat Oncol Biol Phys.

[9] Abugideiri M, Nanda RH, Butker C (2016). Factors influencing pulmonary toxicity in children undergoing allogeneic hematopoietic stem cell transplantation in the setting of total body irradiation-based myeloablative conditioning. Int J Radiat Oncol Biol Phys.

[10] Esiashvili N, Lu X, Ulin K (2019). Higher reported lung dose received during total body irradiation for allogeneic hematopoietic stem cell transplantation in children with acute lymphoblastic leukemia is associated with inferior survival: a report from the children's oncology group. Int J Radiat Oncol Biol Phys.

[11] Vogel J, Hui S, Hua CH (2021). Pulmonary toxicity after total body irradiation - critical review of the literature and recommendations for toxicity reporting. Front Oncol.

[12] Hall MD, Schultheiss TE, Smith DD, Nguyen KH, Wong JY (2015). Dose response for radiation cataractogenesis: a meta-regression of hematopoietic stem cell transplantation regimens. Int J Radiat Oncol Biol Phys.

[13] Shinde A, Yang D, Frankel P (2019). Radiation-related toxicities using organ sparing total marrow irradiation transplant conditioning regimens. Int J Radiat Oncol Biol Phys.

